# Editorial: Redox Biology of Skin Aging and Carcinogenesis: the Role of Natural Antioxidants as Potential Protective Agents

**DOI:** 10.3389/fphar.2020.00249

**Published:** 2020-03-06

**Authors:** Uraiwan Panich, Andrzej T. Slominski

**Affiliations:** ^1^Department of Pharmacology, Faculty of Medicine Siriraj Hospital, Mahidol University, Bangkok, Thailand; ^2^Department of Dermatology, University of Alabama at Birmingham, Birmingham, AL, United States; ^3^Comprehensive Cancer Center, Cancer Chemoprevention Program, University of Alabama at Birmingham, Birmingham, AL, United States; ^4^VA Medical Center, Birmingham, AL, United States

**Keywords:** antioxidant, oxidative stress, photoaging, photocarcinogenesis, ultraviolet

Ultraviolet radiation (UVR) has been generally known to have both beneficial and detrimental effects on human health (Jeayeng et al., 2017; Slominski et al., 2018b). While UVR plays a beneficial role in natural synthesis of vitamin D and proopiomelanocortin (POMC)-derived peptides including endorphins and other neurohormones in the skin (Slominski et al., 2000, 2012), excessive exposure to UVR can lead to acute and chronic adverse effects on the skin and eyes (Green et al., 2011). The harmful effects of UVR on sun-exposed areas of the skin have been intensively investigated and suggested to associate with premature aging (or photoaging) (Bocheva et al., 2019) and increase risk for developing skin cancers. UVR contributes to cutaneous photodamage via various mechanisms including DNA damage, oxidative damage, and inflammation. Oxidative stress can disrupt the homeostasis of skin cells through damage to the DNA, proteins, and interference with cell signaling pathways leading to cell death, apoptosis or malignant transformation (Venza et al., 2015; Chaiprasongsuk et al., 2019). In addition to the UV light having a negative impact on the skin, prolonged exposure of the skin to environmental stressors (physical, chemical, and biological stressors) can lead to structural damage and functional impairment and increased vulnerability to cutaneous diseases and problems including skin cancer. Parrado et al. comprehensively reviewed the molecular mechanisms involved in skin aging mediated by environmental stressors (especially air pollutants and UVR) (Parrado et al.). Exposure of the skin to air pollutants can disrupt skin's function and various biological responses including oxidative stress, DNA damage, mitochondrial damage, apoptosis, inflammation, skin barrier impairment, extracellular matrix disruption, and melanogenesis via interfering with signaling cascades [e.g., the aryl hydrocarbon receptor (AhR) and NF-κB signaling pathways]. This review also addressed a role of synergistic damage by UVR and air pollutants, e.g., particular matter (PM), in premature skin aging.

Due to increased awareness about the harm of UV light, demand continues to grow globally for effective and safe photoprotective agents having UV protection and pharmacological activities against photodamage. This Research Topic includes original research and review articles from experts in this field to discuss the role of natural and synthetic antioxidants in the skin photoprotection that would provide insight into development of effective and safe candidates which might act synergistically with sunscreen-based approaches for prevention and treatment of skin photodamage. The transcription factor nuclear factor erythroid 2-related factor 2 (Nrf2) is the master regulator of the antioxidant response protecting skin cells against oxidative insults (Rojo de la Vega et al., 2017; Battino et al., 2018; Chaiprasongsuk et al., 2019). Various natural and synthetic compounds, particularly electrophiles, having abilities to activate Nrf2-regulated antioxidant defense and promote redox balance have been demonstrated to provide promising photoprotective effects against skin damage. Rojo de la Vega et al. reported the first *in vivo* evidence for Nrf2-dependent skin photoprotection of the apocarotenoid bixin, an FDA-approved food additive and cosmetic ingredient from the seeds of the achiote tree (*Bixa orellana*), in two genetically modified mouse models (SKH1 and C57BL/6J (*Nrf2*^+/+^ vs. *Nrf2*^−/−^). A bixin formulation optimized for topical Nrf2 activation suppressed acute UV-induced photodamage in *Nrf2*^+/+^ but not *Nrf2*^−/−^ SKH1 mice. Topical bixin also exerted Nrf2-dependent protection against hair graying induced by PUVA (psoralen + UVA) in *Nrf2*^+/+^ but not *Nrf2*^−/−^ C57BL/6J mice.

Dunaway et al. provided an in-depth review of photoprotective mechanisms of natural antioxidants against solar UVR-mediated skin aging. Cutaneous compounds [e.g., melatonin and vitamin D (Slominski et al., 2018a; Chaiprasongsuk et al., 2019)] and botanical compounds (e.g., tea polyphenols, grape seed polyphenols, honokiol (*Magnolia Sp*.), sulforaphane, quercetin, apocynin, curcumin, silymarin milk thistle, aloe vera, ginseng, algae, propolis) might exert protective effects on skin photodamage through various mechanisms including anti-inflammatory, immunomodulatory, and detoxifying actions as well as modulation of antioxidant defense and gene expression (Dunaway et al.). The hallmarks of UVR-induced skin aging include stimulation of matrix-metalloproteinases (MMPs) accountable for degradation of extracellular matrix of the dermis, in particular, collagen as well as a compromised dermoepidermal junction. Thus, several naturally derived compounds acting as anti-photoaging agents are capable of downregulating MMPs including MMP-1 (interstitial collagenase), MMP-3 (stromelysin-1) and MMP-9 (gelatinase-B) (Bae et al., 2016; Pittayapruek et al., 2016; Chaiprasongsuk et al., 2017). Carpenter et al. reported potential photoprotective effects of the meadowfoam plant (*Limnanthes alba*) glucosinolate derivatives, 3-methoxybenzyl isothiocyanate (MBITC) and 3-methoxyphenyl acetonitrile (MPACN), on UVB-induced DNA damage, MMP-1 and MMP-3 expression as well as epidermal proliferation and thickness in three-dimensional human skin reconstructed *in vitro*, indicating their roles to prevent photoaging and photocarcinogenesis (Carpenter et al.). Additionally, Przystupski et al. demonstrated that natural compounds with antioxidant activity including catechin isolated from green tea, honokiol derived from magnolia, curcumin from turmeric and cinnamon extract potentially mitigated deleterious impact of stratospheric environment on stress responses (including apoptosis, oxidative stress, and DNA damage) in human normal and cancer cells (Przystupski et al.). Biological responses to radiation and antioxidants varied in different cell types. Catechin and honokiol were observed to provide the greatest radioprotective effect on normal cells whereas curcumin and cinnamon had potential photosensitizing effect on cancer cells.

Since the role of UVR-induced oxidative stress in photoaging involves damage to mitochondria, which is susceptible to ROS, mitochondrially targeted agents to reduce mitochondrial stress and subsequent DNA damage could serve as a promising photoprotection strategy.

Finally, Brand et al. reviewed the evidence of the naturally occurring compounds and synthetically generated mitochondrial targeted cyclic nitroxides as promising alternatives for prevention and reduction of skin photodamage (Brand et al.). It is crucial to design synthetic antioxidants which can reach their mitochondrial target in adequate concentrations to achieve the effect.

In conclusion, this Research Topic provides updated studies and reviews providing insights into cellular and molecular mechanisms by which natural and synthetic antioxidants mitigate skin damage induced by environmental stressors including UVR. Although development of appropriate formulation supported by clinical evidence is required in order to ensure effectiveness and safety of a product, compound candidates with antioxidant and photoprotective properties deserve further development as potential pharmacological strategies for prevention and treatment of skin aging (Bocheva et al., 2019) and cancers.

## Author Contributions

All authors listed have made a substantial, direct and intellectual contribution to the work, and approved it for publication.

### Conflict of Interest

The authors declare that the research was conducted in the absence of any commercial or financial relationships that could be construed as a potential conflict of interest.
